# Design of a Nutritional Survey to Detect High Dietary Salt Intakes and Its Usefulness in Primary Care Compared to 24-Hour Urine Sodium Determination

**DOI:** 10.3390/nu15061542

**Published:** 2023-03-22

**Authors:** Amelia Jiménez Rodríguez, Luis Palomo Cobos, Amelia Rodríguez-Martín, Patricia Fernández del Valle, José P. Novalbos-Ruíz

**Affiliations:** 1 Centro de Salud de Alcuéscar, 10160 Cáceres, Spain; 2 Centro de Salud de Zona Centro, 10001 Cáceres, Spain; 3 Departamento de Biomedicina, Biotecnología y Salud Pública, Universidad de Cádiz, 11003 Cádiz, Spain; 4 Complejo Hospitalario Universitario de Cáceres, 10004 Cáceres, Spain

**Keywords:** nutritional survey, high dietary salt, dietary salt intake, urine sodium, primary health care, urban

## Abstract

Many population studies report salt intakes that exceed the WHO recommendation (2 g/day of Na+ or 5 g/day of salt). We do not have tools for detecting high salt intakes that are easy to apply in primary health care (PHC). We propose the development of a survey to screen for high salt intake in PHC patients. A cross-sectional study of 176 patients determines the responsible foods, and a study of 61 patients studies the optimal cut-off point and discriminant ability (ROC curve). We assessed the salt intake using a food frequency questionnaire and a 24 h dietary recall and used a factor analysis to identify the foods with the highest contribution to be included in a high intake screening questionnaire. We used 24 h urinary sodium as a gold standard. We identified 38 foods and 14 factors representing a high intake, explaining a significant proportion of the total variance (50.3%). Significant correlations (r > 0.4) were obtained between nutritional survey scores and urinary sodium excretion, allowing us to detect patients who exceed salt intake recommendations. For sodium excretion ≥ 2.4 g/day, the survey has a sensitivity of 91.4%, a specificity of 96.2% and an area under the curve of 0.94. For a prevalence of high consumption of 57.4%, the positive predictive value (PPV) was 96.9% and the negative predictive value (NPV) was 89.2%. We developed a screening survey for subjects with a high probability of high salt intake in primary health care, which could contribute to the reduction in diseases associated with this consumption.

## 1. Introduction

According to the INTERSALT study, high salt intakes are closely related to increased blood pressure, and low intakes lead to lower blood pressure levels [1].

The scientific evidence for the role of salt in the pathogenesis of hypertension has been confirmed by numerous studies [2,3]; after the age of 50, almost 50% of the population suffers from hypertension, and between 13% and 16% of all deaths are attributed to it. According to the WHO, hypertension is responsible for at least 45% of deaths due to heart disease, and 51% of deaths due to stroke [4]. Meta-analyses of randomized trials have shown that reducing salt intake by 6 g/day would reduce the incidences of stroke by 24% and coronary heart disease by 18%, preventing more than 2.5 million deaths worldwide from stroke and cardiovascular events [5]. However, the recommendation to reduce the salt intake in patients with hypertensive or cardiovascular disease, which is so common in primary care, is rarely supported by the prior use of food frequency questionnaires to identify the consumption of high salt foods.

Although an excessive dietary salt intake is clearly related to different causes of morbidity, such as hypertension, cardiovascular disease (CVD), overweight, osteoporosis, or gastric cancer [6], it is not common for primary care, hospital medical, or nursing practices to use methods to reliably measure the degree of the salt intake of patients. Enquiries about a high salt intake dietary pattern do not go beyond generic questions, and as a result, recommendations to reduce salt intake in hypertensive or cardiovascular patients are rarely based on knowledge of the patient’s diet and are not accompanied by documentation or other tools to identify foods with a high salt content.

The WHO recommends reducing one’s dietary salt intake as a cost-effective strategy to reduce blood pressure and the risk of CVD, stroke, and coronary heart disease [7]. This recommendation is strong when the daily salt intake in adults is higher than 5 g/day [8] according to the WHO, or 5.8 g of salt in the US dietary recommendations [9]. The problem for clinicians is how to identify patients who exceed these dietary salt limits.

The standard method for assessing salt intake is to measure 24 h of urinary sodium excretion, which is rarely used in primary health care due to its cumbersome nature. To simplify the measurement, equations such as those of Tanaka [10] and Kawasaki [11] or the INTERSALT equation [12] are used, which have been used to quantify sodium intake from sodium excretion in fractional or spot urine samples at certain times of the day [10]. Another way to measure salt intake is through the use of food questionnaires, such as food frequency questionnaires of the most and least salt-rich foods, or 24 h food diaries that are spread over several days. However, these consumption surveys often include all types of foods, without differentiating according to their Na+ contribution, making it risky to make dietary recommendations that discriminate between foods with the highest salt intake [13].

The aim of this study is to develop a nutritional survey that will allow us to easily identify individuals with high salt intakes in primary care consultations and to determine an optimal cut-off point from which to detect patients who exceed the recommended limits, based on a broad list of foods present in the usual diet of Spanish adults that have been shown to correlate with 24 h urinary salt excretion. We analyze the predictive value of high intakes for the different cut-off points proposed in international recommendations.

## 2. Materials and Methods

### 2.1. Design of Nutritional Survey

The food items included in the survey were obtained from a cross-sectional observational study of a normotensive and hypertensive population sample, in which total daily salt intake, estimated from 24 h urinary sodium excretion (gold standard), and behaviors related to salt addition were determined. The origin, method of recruitment, and characteristics of the sample of 176 participants are described in a previous study [14]. In summary, participants belonged to an urban health center (Cáceres, Spain), were aged 46–75 years, were consecutively invited to participate, and underwent an interview and a physical examination including measurement of blood pressure, abdominal circumference, BMI, blood, and 24 h urine collection. Blood parameters included the following, among others: Na+ (sodium), K+ (potassium), glucose, cholesterol, urea, creatinine, and GFR (glomerular filtration rate). In urine we also determined albuminuria, albumin/creatinine ratio, creatinine, and glucose. The research protocol was favorably evaluated and approved by the Biomedical Research Ethics Committee of the Health Council. All participants signed the informed consent form.

All participants were asked to complete two food surveys: a food consumption frequency questionnaire (FFQ) validated in the adult population [15], and a 24 h recall nutritional survey, which included questions of typical foods of the Extremadura region with high Na+ content, as well as behaviors related to the addition of salt in food preparation and the use of salt shakers at the table. The sodium content of the food was extracted from the food composition table (BEDCA network) [16]; the nutritional assessment was performed based on the intake of the recall of food ingested in the last 24 h assessed with the EvalFINUT program of the Ibero-American Nutrition Foundation [17].

Study participants collected a 24 h urine sample on the same day as the nutritional assessments, so we studied the correlations between estimated salt intake from the surveys and that determined from 24 h urinary sodium excretion. We consider the presence of Na+ in 24 h urine between 2.07 and 5.05 g/L/day (equivalent to 90–220 mEq/L/day) as reference values. To assess the completeness of 24 h urine collections, we included self-report and a 24 h urine volume and assessment of 24 h creatinine excretion based on calculations using age, sex, and weight.

The same study [14] described the characteristics of the average salt intake found in the sample (6.6 g in men and 7.5 g in women) and identified the foods with the highest salt contribution to the participants’ diets based on correlations between reported intake in surveys and 24 h urinary sodium excretion.

With these foods identified, using an exploratory factor analysis, we developed a specific nutritional survey to screen for high salt intakes. The factor analysis allows for data reduction and finding homogeneous groups of foods rich in Na+ with higher intakes, which represent the dietary profile of patients with high salt intakes.

Reliability (internal consistency) was assessed using Cronbach’s alpha test, with values ranging from 0 to 1; values α > 0.70 were considered acceptable, and α > 0.80 good. Construct validity was determined by exploratory factor analysis; sample adequacy was assessed by applying the Kaiser-Meyer-Olkin (KMO) test, with values greater than 0.5, and Bartlett’s test of sphericity, with significant values. To determine unidimensionality, the following criteria were taken into account: (1) that all items had a Pearson’s r > 0.30 in the first factor during extraction; (2) that the first factor explained a significant proportion of variance with respect to the other factors; and (3) that the total variance explained by the main extracted factors was greater than 50%.

Varimax rotation was used to minimize the number of foods and determine which foods had high loadings for each factor. To include an item in the orthogonal factors, values with a Pearson’s r > +0.40 were considered relevant.

### 2.2. Survey Validity, Optimal Cut-Off, and Discriminant Ability

A frequency of consumption survey with the selected foods was created from the results of the factor analysis, using an odd 7-point Likert-type scale, with 1 representing never and 7 representing more than once a day (see Supplementary Materials File). The scoring method was the sum of the individual items. This new survey was applied to a new sample of 61 participants with the same selection criteria and in the same primary care centers, and we collected variables such as age, sex, weight, height, diseases and comorbidities, and whether or not they were on a diet for any reason. At the same time, 24 h of urine was collected from the participants to estimate their actual salt intake.

To determine an optimal cut-off point in the questionnaire score to help identify subjects whose salt intake can be considered high, ROC curves were generated, using as possible cut-off points those that maximize sensitivity and specificity (maximum Youden index) for the recommended high salt intake endpoints of 3 g/day and 2.4 g/day. Sensitivity, specificity, positive predictive value (PPV), and negative predictive value (NPV) were estimated for each of these cut-off points, and the area under the curve (AUC) was calculated as an index of accuracy.

We then used logistic regression to analyze the probability of high sodium intake detected by the questionnaire according to the different recommended values (2.4 and 3 g/day Na+) adjusting the results for age, sex, and BMI.

## 3. Results

A total of 176 patients participated in the study, with a mean age of 62 (SD 8) years and a slight female predominance (53.4%). A total of 32.4% have experienced higher education; 43.7% are employed; and 69.9% perform physical exercise, of which 67.9% reach the recommendations of 150 min a week. A total of 78.4% are overweight and obese (BMI ≥ 25), and 61.15% report eating out at least once a week. A total of 61.4% of patients reported drinking alcoholic beverages and only 16.5% were smokers.

With regard to the main clinical characteristics of interest for this study, almost 60% of the study participants had hypertension, 42% had dyslipidemia, and 17% had diabetes mellitus; most of the patients were treated for these chronic conditions. The average Na+ intake estimated using the food frequency questionnaire (FFQ) was around 2.5 g; the Na+ intake determined by the FFQ typical of the local Extremaduran diet provided an approximate value of 3 g in both men and women. From the 24 h dietary recall nutritional survey, we obtained a total dietary Na+ intake of 6.6 g in men and 7.5 g in women. We found a good correlation between the Na+ estimated in the generic food consumption frequency questionnaire and the questionnaire assessing the subset of foods in the local diet.

The perception of salt consumption and its use at the table was also asked; 56.3% think they have an “adequate” salt consumption while 39.8% think their salt consumption is “low”. A total of 32.4% say that they add salt to food once it is at the table and 56.9% of patients add more salt while preparing food in the kitchen.

The total 24 h urinary Na+ excretion in the population is 3.7 (SD 1.4) g and is significantly higher in men (4.2 g/day vs. 3.2 g/day in women). Urine Na+ levels are higher than the recommended 2 g in 92% of the subjects; if we use 2.5 g/day as a cut-off point, this percentage would decrease to 75%. If we classify sodium intake on the basis of the Na+/K+ ratio and consider high sodium intake when its absolute value is above 1, the percentage of subjects in the sample with high sodium intake is 79.54%. Considering the levels of salt intake as determined by 24 h urine and the perception of consumption expressed by the patients, almost 84% of patients with elevated Na+ excretion levels are not aware that they are consuming more salt than recommended.

When we analyzed the correlation between Na+ intake estimated using the different questionnaires and the values determined in 24 h urine (gold standard), Na+ estimated using both instruments had a very weak significant correlation with urinary Na+ excretion.

On the basis of the correlations between the intakes obtained from the food consumption frequency questionnaires and the 24 h urinary sodium excretion, we identified the foods that, due to their frequency of consumption and salt content, could represent a higher contribution of salt to the diet of the participants.

The questionnaire was designed using the 48 foods that were highly correlated with salt intake (Supplementary Table S1). Cronbach’s alpha showed an overall internal consistency of 0.221 and after exploratory factor analysis, 10 foods were removed. The final version had a Cronbach’s alpha of 0.926. The sample adequacy study showed a KMO of 0.636 and Bartlett’s test was statistically significant. The factor analysis grouped the 38 foods into 14 factors with an eigenvalue greater than or equal to 1. This result explained a significant percentage of the total variance (50.30%) (Table 1).

The resulting questionnaire (Supplementary Table S3) was administered to a sample of 61 patients, aged 46–75 years, who signed and accepted the informed consent form, with Spanish nationality or with more than 25 years of residence in Spain (assuming they followed a similar Mediterranean dietary pattern), without eating disorders or severe chronic kidney disease, and selected consecutively in several primary care consultations in the Cáceres health area. These participants were asked about the frequency of consumption of these foods in the previous month, using 7-point Likert scale. The subjects had a mean age of 58.6 years (SD 7.8); 47.5% were women and the mean score obtained was 128.8 points (SD 7.82) (Table 2). In Table 2, we can observe the average Na+ intake of the subjects by 24h urine excretion, showing levels above the recommended levels. The sample of subjects had a mean excretion of 3.14 g/day, being able to distinguish two main cuts; a total of 35 subjects with ≥2.4 and about 27 subjects with equivalent levels ≥ 3 g.

**Figure 1:**
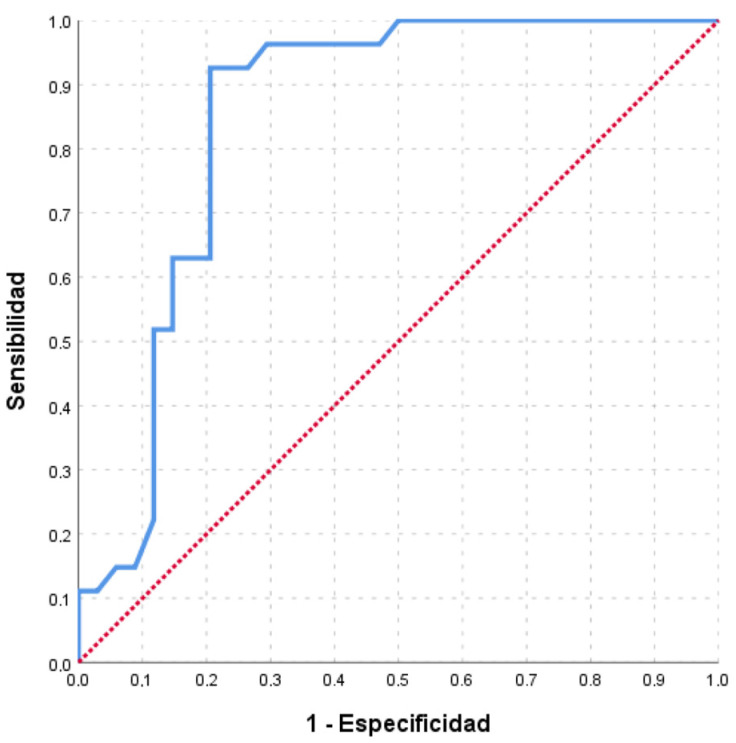
ROC curves of the scores obtained in the survey to estimate sodium intakes greater than 3 g/d (P3000). Area under the curve (AUC) = 85.0% (95% CI: 74.8–95.2).

**Figure 2:**
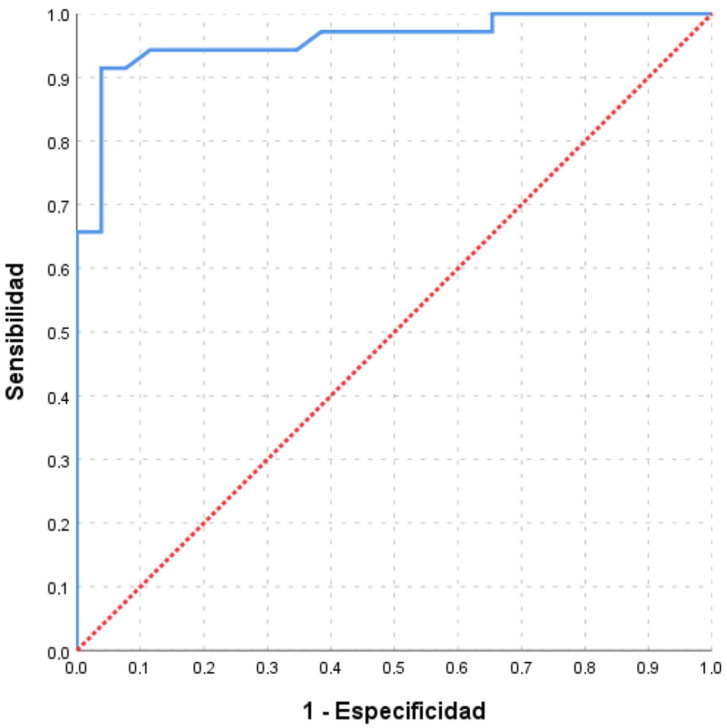
ROC curves of the scores obtained in the survey to estimate sodium intakes greater than 2.4 g/d (P2400). Area under the curve (AUC) = 95.8% (95% CI: 91.0–100).

The points on the ROC curves (Figure 1 and Figure 2) providing the highest Youden index for classifying subjects with sodium excretion greater than 3 and 2.4 g/day are a score of 126 and 124 points, respectively. The optimal cut-off point for detecting patients with salt intake above 3 g/day, set at 126 points, estimated a sensitivity of 92.59% (95% CI: 80.86–100), specificity of 79.41% (95% CI: 64.35–100), PPV of 78.13% (95% CI: 62.24–100), and NPV of 93.10% (95% CI: 82.16–100); while for the cut-off point corresponding to ≥2.4 g/day, 124 points or more, the sensitivity was 91.43% (95% CI: 80.73–100), the specificity was 96.15% (95% CI: 86.84–100), the PPV was 96.97% (95% CI: 89.61–100), and the NPV was 89.29% (95% CI: 76.04–100) (Table 3).

Table 3 shows the logistic regression analysis, where it is observed that the cut-off point of 124 points is significantly associated with a higher probability of having a urinary sodium excretion ≥ 2.4 g/day (OR: 269.9; 95% CI 21.4–3402.2), whereas with the cut-off point of 126 points the probability of having a urinary sodium excretion ≥ 3 g/day, is somewhat lower (OR: 66.4; 95% CI 9.1–484.5).

## 4. Discussion

Increased urinary sodium excretion, representing dietary sodium intake, is associated with hypertension. In a dose–response meta-analysis assessing the relationship between sodium intake (estimated from dietary intake or urinary excretion) and risk of hypertension in cohort studies, an almost linear relationship between sodium intake/excretion and hypertension risk was found, with an excess risk starting at 3 g/day [18]. Other studies have demonstrated that due to the J-shaped association of sodium intake with plasma renin activity and systolic blood pressure, the risk of mortality and cardiovascular events increases when intake exceeds 5 g/day [19,20].

Meta-analyses demonstrate that a reduction in dietary sodium intake according to public recommendations is associated with an average reduction in systolic/diastolic blood pressure of 5.7/2.9 mm Hg in hypertensive subjects [21]. In people with normal blood pressure, the effects of sodium reduction were more consistent on potential side effects (hormones and lipids) than the effect on blood pressure. This review reinforces the validity of recommendations to prevent cardiovascular disease by reducing sodium intake in hypertensive adults.

Worldwide, less than 5–10% of people consume less than 2.3 g/day of sodium, but it is difficult to estimate this intake accurately because the salt content of meals is uncertain. We have found that patients underestimate the amount of salt in food and salt added in cooking or at the table; hence, the best method to determine salt consumption is by quantifying the amount excreted in 24 h urine. However, the determination of Na+ in 24 h urine as a gold standard for the detection of patients with high intakes is rarely used because of its complicated collection and cost. Instead, many studies propose the use of fractionated urine samples or standardized questionnaires to quantify the frequency of consumption of foods with a higher salt content [22,23,24].

Considering urinary Na+ excretion as a reference value, when we estimate total dietary Na+ intake from the analysis of those collected in the 24 h reminder nutritional survey, we tend to overestimate intake (we obtained 7.25 g/day, which almost doubles the urine values), while the generic food frequency questionnaire underestimates it [22]. Malavolti et al. [25] estimated dietary Na+ and K+ intakes in 719 Italian adults using the FFQ; the mean sodium intake was estimated at 2.15 g/day while the mean potassium intake was 3.37 g/day; these values are very similar to those obtained in our participants using the generic food frequency questionnaire, which underestimates intake (compared with 24 h urine collections). The foods that contributed most to sodium intake were cereals, meat products (especially processed meat), and dairy products, and for potassium, they were red and white meats, fresh fruit, and vegetables; these are many convenience and processed foods (industrialized countries) and few related to local dishes (pickles, sausages, cheese, and salted fish in Mediterranean countries) [24,25,26,27]. Following the recommendations of similar studies [26,27,28], we contrasted Na+ intake using two food frequency questionnaires and nutritional assessment with a 24 h recall nutritional survey. We found greater validity in the quantification of Na+ intake using specific salt-rich foods questionnaires, such as the one we propose.

At present, there is no specific questionnaire for the detection of subjects with high salt intake adapted to our setting that can be easily applied in primary care to hypertensive patients and/or those with associated comorbidities. Most of the studies we reviewed on food questionnaires did not categorize foods according to the amount of salt they contain according to the food composition table [11,15,29] or their contribution to total salt intake.

Several authors [27,30] agree on the need for an adapted dietary questionnaire that would allow us to assess the salt intake of patients who are eligible for intervention in the short or medium term, as this would facilitate the restriction of salt in the diet of our patients and the implementation of preventive educational and dietary interventions [22]. In our study, we designed a food consumption frequency questionnaire in which we basically assessed the intake of a set of foods that represent a significant contribution of salt to the diet, either because of their sodium content or because of the combination of their sodium content and the amount of their intake, not with the aim of obtaining a precise estimate of the amount of salt, but rather to detect subjects with a high intake.

Dietary variability is described according to the community and/or city in Spain where each participant resides, but no great variations are obtained. For example, in a study carried out in seven regions in cities such as La Coruña, Barcelona, Burgos, Palma de Mallorca, Pamplona, Valencia, and Zaragoza, the diet was collected by means of a food frequency questionnaire validated for the Spanish population [31]. All those interviewed had a high intake of dairy products and pulses, and fruit and vegetables had a high intake in Mallorca and Valencia, whereas it was low in La Coruña. Olive oil consumption was high in all places except Burgos, where 74.3% of the women studied were below the recommended three servings per day. As a result of this study, an insufficient intake of vitamin E was found in La Coruña and Burgos. Therefore, we can observe that dietary peculiarities were only found in areas far from the coast with a higher consumption of dairy products. There are regional variations in the consumption of certain foods; for example, in Spain, more fish is generally eaten in southern areas than in central areas, more meat in the inland, and more vegetables in the eastern regions [31].

As a proposed screening tool, the questionnaire we have developed is not intended to quantify the salt intake precisely, but to detect subjects with a high probability of high salt intake. The sample from which the initial list of foods with a significant contribution to salt intake was drawn [14] and the sample in which the survey was tested are similar in terms of the most common epidemiological variables such as age, sex, comorbidities, height, and weight. The foods included in the questionnaire are routine foods in our Mediterranean diet, in any Spanish region. However, we have included in the questionnaire some foods that are not so commonly consumed outside the region of Extremadura (such as some Extremaduran cheeses or paprika as an additive), but that are useful to give more strength to the questionnaire.

The survey elaborated on the frequency of consumption of foods with a high Na+ intake in the diet, which leads to determining a modifiable risk in patients. It a novel survey that is easy to apply (no more than 10 min, taking into account that we also ask about pathology and diet and record anthropometric measurements) and very useful and essential for primary care teams. In this way, we would only have to interview our patients for a few minutes in the consulting room, and according to the score obtained in the nutritional survey and the determination of anthropometric parameters, we could act accordingly on the estimated risk associated with high Na+/salt intake.

Once this questionnaire has been completed and verified, its usefulness for the follow-up of patients in whom we are going to intervene with dietary salt restriction would need to be tested. In these patients, the questionnaire would help to focus on which foods to intervene with, and the sensitivity to change the questionnaire could help in assessing compliance with our recommendations in the primary care office.

These results suggest that a nutritional survey consisting of a combined food consumption and food frequency questionnaire could be valid for the identification of populations with high salt intakes. Food frequency questionnaires allow us to obtain information on the pattern of usual consumption in different populations. The use of food frequency questionnaires is an applicable methodology that is easy, quick, and less costly. In addition, they involve less effort for the interviewed subjects than invasive/non-invasive tests, such as 24 h blood or urine samples [28].

Although there are doubts about the beneficial effects of reducing salt intake in populations with moderate intake levels [19], a linear relationship between all levels of salt intake and the incidence of hypertension, cardiovascular disease [18], and all-cause mortality [32] seems to have been demonstrated. In conclusion, we provide a simple tool that allows us to detect subjects with a high salt diet, which is easy to use in primary health care and could reduce costs and save time compared to the current gold standard of 24 h urine. Taking into account the quantification of dietary intake obtained in this study, a total score of 124 points or higher regardless of the age, sex, and BMI of the subjects is significantly associated with a higher likelihood of having a sodium excretion ≥ 2.4 g/day and, therefore, a salt intake above the recommendations.

## Figures and Tables

**Table 1:** Psychometric characteristics of the questionnaire.

	Cronbach’s α	KMO *	Bartlett’s Test of Sphericity	No. of Factors	% Explained Variance
48 foods	0.221	0.594	0.000	18	50.7%
38 foods	0.926	0.636	0.000	14	50.3%

* KMO: Kaiser–Meyer–Olkin.

**Table 2:** Characteristics of the sample.

		Total (*n* = 61)
Gender	Men *n* (%)	32 (52.5)
	Women *n* (%)	29 (47.5)
Average age (SD) *		58.57 (7.8)
Comorbidities **	HBP *n* (%)	22 (36.1)
	AODM *n* (%)	14 (23.0)
	Dyslipidemia *n* (%)	24 (39.3)
	BMI ≥ 25 *n* (%)	35 (57.4)
Average mark (SD)		128.8 (18.6)
Diets	HBP	9 (14.7)
	AODM	8 (13.1)
	DLP	12 (19.7)
	CKD	1 (1.6)
	Weight loss	16 (26.22)
Na excretion + urine 24 h	Grams/day (SD)	3.14 (1.7)
	≥2.4	35 (57.4)
	≥3	27 (44.3)

* SD: standard deviation. ** Comorbidities. HBP: high blood pressure; AODM: adult onset diabetes mellitus 2; DLP: dyslipidemia; CKD: chronic kidney disease.

**Table 3:** Sensitivity, specificity, and Youden index for intakes above the cut-off points.

	Cut-Off Point	Sensitivity	Specificity	Youden Index	PPV *	NPV
P2400 **	124	91.44%	96.15%	0.88	96.97%	89.29%
P3000 ***	126	92.59%	79.41%	0.72	85.25%	78.13%

* PPV: positive predictive value, NPV: negative predictive value. ** P2400: Sodium intakes above 2.4 g/day determined in 24 h urine. *** P3000: Sodium intakes above 3 g/day determined in 24 h urine.

## Data Availability

The data presented in this study are available on request from the corresponding author.

## Supplementary Materials

The following supporting information can be downloaded at: https://www.mdpi.com/article/10.3390/nu15061542/s1, Supplementary Table S1: Foods highly correlated with salt intake; Supplementary Table S2: Present comorbidities and monitoring of diets. Supplementary Table S3: Nutritional Survey for the detection of high salt intakes.
