# Supplementary material for: Design of a Nutritional Survey to Detect High Dietary Salt Intakes and Its Usefulness in Primary Care Compared to 24-Hour Urine Sodium Determination
Source: Nutrients. 2023 Mar 22;15(6):1542. doi: 10.3390/nu15061542 (PMC10056562; doi:10.3390/nu15061542)
Supplement: Supplementary file 1 [file nutrients-15-01542-s001.zip › nutrients-2256578-supplementary.pdf]

Table S1.

Foods highly correlated with salt intake.

FOODS
Milk (1 glass or cup, 200 cc)
Skimmed yogurt (one, 125 grams)
Cottage cheese, white or fresh cheese (one serving or serving)
Cured, semi-cured, or creamy cheese (one piece)
Cured Extremaduran cheeses: Ibores, Casar (piece, portion)
Eggs (1 egg)
Game meat (medium dish)
Ham (2 strips)
Chicken (1 medium plate or piece)
Veal, pork, lamb as main course<br>(1 medium plate or piece)
Sausages: sausage, salami, mortadella (50 g serving)
Sausages and the like (one medium)
Pâtés, foie gras (half serving)
Bacon, bacon, bacon (2 strips or slices)
Assorted fried fish (1 medium dish or serving)
Boiled or grilled fish: hake, sole, sea bream, emperor, salmon, bonito, tuna(1 dish or serving)
Other oily fish: mackerel, sardines, anchovy/anchovies
A small can of preserves: tuna, bonito, sardines, mackerel
Salted and/or smoked fish: anchovies, cod<br>(half serving)
Cooked spinach or chard (1 medium dish)
Cabbage, cauliflower, cooked broccoli (1 medium dish)
Artichokes (1 medium plate, 1 tin)
Lettuce, endive, endive (1 medium dish)
Tomato (one medium)
Onion (one medium)
Carrot, pumpkin (one or small plate)
Cooked green beans (1 course)
Peppers (one)
Legumes: lentils, chickpeas, pinto or white beans, cooked, stew (1 medium dish)
Soup or vegetable puree (a dish)
Chicken croquettes, ham (one)
Fried fish sticks or delicacies (one)
White bread (One small piece/3 mold slices/60 g)
French fries (1 serving or plate)
Boiled, roasted potatoes (1 medium potato)
Potato chip bag (1 small bag)
Cooked rice (1 medium dish)
Pasta: spaghetti, noodles, macaroni and similar (1 dish)
Pizza (1 slice or serving)
Vegetable oil/olive (1tablespoon)
Cookies: Maria type, chocolate (1 cookie)
Pastries: croissant, donut, muffin, sponge cake, cake or similar (one or portion)
Beer (one cane or bottle 1/5, 200 cc)
Olives (a plate or lid of about 15 small units)
Nuts: almonds, peanuts, pine nuts, hazelnuts (1 plate or sachet, 30g)
Salt added to dishes on the table (1 pinch or pinch)
Paprika (1 pinch or a pinch)
Mayonnaise (1 tablespoon)
Ketchup (1spoon)

Table S2

Present comorbidities and monitoring of diets.

Point out if you have any of the following **conditions or diseases**	Are you following any "slimming **regimen/weight loss diet" or "special diets for medical conditions"**? Point out the **reason** (you can check more than one)
○ DIABETES<br>○ HIGH CHOLESTEROL<br>○ HYPERTENSION<br>○ KIDNEY DISEASE<br>○ DIURETICS	○ NO<br>○ YES<br>○ To control my blood sugar level<br>○ Because of cholesterol<br>○ For hypertension<br>○ For my kidney disease<br>○ To control weight

Table S3. ***Nutritional Survey for the detection of high salt intakes.***

FOODS	NEVER	2-3 DAYS PER MONTH	1 DAY A WEEK	2-4 DAYS A WEEK	5-6 DAYS A WEEK	1 TIME A DAY	MORE THAN 1 TIME DAY
Milk (1 glass or cup, 200 cc)							
Skimmed yogurt (one, 125 grams)							
Cottage cheese, white or fresh cheese (one serving or serving)							
Cured, semi-cured, or creamy cheese (one piece)							
Cured Extremaduran cheeses: Ibores, Casar (piece, portion)							
Eggs (1 egg)							
Game meat (medium dish)							
Ham (2 strips)							
Chicken (1 medium plate or piece)							
Veal, pork, lamb as main course (1 medium plate or piece)							
Sausages: sausage, salami, mortadella (50 g serving)							
Sausages and the like (one medium)							
Pâtés, foie gras (half serving)							
Bacon, bacon, bacon (2 strips or slices)							
Assorted fried fish (1 medium dish or serving)							
Boiled or grilled fish: hake, sole, sea bream, emperor, salmon, bonito, tuna(1 dish or serving)							
Other oily fish: mackerel, sardines, anchovy/anchovies							
A small can of preserves: tuna, bonito, sardines, mackerel							
Salted and/or smoked fish: anchovies, cod (half serving)							
Cooked spinach or chard (1 medium dish)							
Cabbage, cauliflower, cooked broccoli (1 medium dish)							
Artichokes (1 medium plate, 1 tin)							
Lettuce, endive, endive (1 medium dish)							
Tomato (one medium)							
Onion (one medium)							
Carrot, pumpkin (one or small plate)							
Cooked green beans (1 course)							
Peppers (one)							
Legumes: lentils, chickpeas, pinto or white beans, cooked, stew (1 medium dish)							
Soup or vegetable puree (a dish)							
Chicken croquettes, ham (one)							
Fried fish sticks or delicacies (one)							
White bread (One small piece/3 mold slices/60 g)							
French fries (1 serving or plate)							
Boiled, roasted potatoes (1 medium potato)							
Potato chip bag (1 small bag)							
Cooked rice (1 medium dish)							
Pasta: spaghetti, noodles, macaroni and similar (1 dish)							
Pizza (1 slice or serving)							
Vegetable oil/olive (1tablespoon)							
Cookies: Maria type, chocolate (1 cookie)							
Pastries: croissant, donut, muffin, sponge cake, cake or similar (one or portion)							
Beer (one cane or bottle 1/5, 200 cc)							
Olives (a plate or lid of about 15 small units)							
Nuts: almonds, peanuts, pine nuts, hazelnuts (1 plate or sachet, 30g)							
Salt added to dishes on the table (1 pinch or pinch)							
Paprika (1 pinch or a pinch)							
Mayonnaise (1 tablespoon)							
Ketchup (1spoon)							
